# A replication study, systematic review and meta-analysis of automated image-based diagnosis in parkinsonism

**DOI:** 10.1038/s41598-022-06663-0

**Published:** 2022-02-17

**Authors:** Paraskevi-Evita Papathoma, Ioanna Markaki, Chris Tang, Magnus Lilja Lindström, Irina Savitcheva, David Eidelberg, Per Svenningsson

**Affiliations:** 1 Department of Clinical Neuroscience, Karolinska Institutet, Stockholm, Sweden; 2 Department of Neurology, Danderyd’s Hospital, Stockholm, Sweden; 3 Center of Neurology, Academic Specialist Center, Torsplan, Box 45436, 10431 Stockholm, Sweden; 4 Center for Neurosciences, The Feinstein Institute for Medical Research, Manhasset, NY USA; 5 Karolinska Institutet, Stockholm, Sweden; 6 Department of Nuclear Medicine, Karolinska University Hospital, Stockholm, Sweden; 7 Department of Neurology, Karolinska University Hospital, Stockholm, Sweden

**Keywords:** Motor control, Basal ganglia, Neuroscience, Biomarkers, Diagnostic markers, Neurology, Parkinson's disease, Medical research, Diagnostic markers

## Abstract

Differential diagnosis of parkinsonism early upon symptom onset is often challenging for clinicians and stressful for patients. Several neuroimaging methods have been previously evaluated; however specific routines remain to be established. The aim of this study was to systematically assess the diagnostic accuracy of a previously developed ^18^F-fluorodeoxyglucose positron emission tomography (FDG-PET) based automated algorithm in the diagnosis of parkinsonian syndromes, including unpublished data from a prospective cohort. A series of 35 patients prospectively recruited in a movement disorder clinic in Stockholm were assessed, followed by systematic literature review and meta-analysis. In our cohort, automated image-based classification method showed excellent sensitivity and specificity for Parkinson Disease (PD) vs. atypical parkinsonian syndromes (APS), in line with the results of the meta-analysis (pooled sensitivity and specificity 0.84; 95% CI 0.79–0.88 and 0.96; 95% CI 0.91 –0.98, respectively). In conclusion, FDG-PET automated analysis has an excellent potential to distinguish between PD and APS early in the disease course and may be a valuable tool in clinical routine as well as in research applications.

## Introduction

Idiopathic Parkinson’s disease (PD) is the most common cause of parkinsonism, a term that reflects neurological disorders with Parkinson-like motor symptoms such as slowness of movement, tremor and rigidity^1^. Less common causes comprise atypical parkinsonian syndromes (APS), including multiple system atrophy (MSA), progressive supranuclear palsy (PSP), corticobasal syndrome and Lewy body dementia^1^. Differential diagnosis of parkinsonism remains challenging for clinicians as PD and APS share several clinical features, especially early on the disease onset. Despite advances in neuroimaging, genetic and biofluid-based biomarkers, the diagnoses are mainly based on clinical criteria^2–5^. Typical signs of each APS are usually mild in the early stages of the disease, which poses difficulties in distinguishing them from PD and among each other^6^. Earlier studies have shown that 75–90% of the patients with PD diagnosis given by a movement disorder specialist had consistent histopathological findings post-mortem^7,8^, whereas a more recent study suggested a clinical accuracy of merely 53%^9^.

Several neuroimaging methods have been successfully evaluated in the differential diagnosis of parkinsonism^10,11^, however specific routines for clinical and research use are not yet firmly established. Brain-circuit abnormalities in PD and other neurodegenerative diseases have been studied through the application of network analysis on ^18^F-fluorodeoxyglucose positron emission tomography (FDG-PET) imaging data^12^, to measure system-related progression and treatment response. Spatial covariance analysis of resting-state metabolic images has been used to identify highly reproducible, disease-specific metabolic brain patterns in PD, MSA, PSP, and corticobasal syndrome^13–15^. Using these patterns, an automated probabilistic two-level algorithm has been developed and validated in an American cohort^16^, and subsequently replicated in other cohorts^17,18^, showing better diagnostic accuracy than clinical assessment by general neurologists and supported by neuropathological results in another cohort^19^.

The aim of this project was to assess the ability of this classification model to distinguish between PD, MSA, and PSP in an unpublished patient series in Stockholm, Sweden, and conduct a meta-analysis in order to synthesize and quantify existing evidence on the diagnostic accuracy of the model.

## Methods

### Ethics

All procedures were in accordance with the ethical standards of the institutional and national research committee and with the 1964 Helsinki Declaration and its later amendments or comparable ethical standards. The study was approved by the Regional Ethical Committee in Stockholm (Decision numbers 2016/19-31/12 and 2019-04967). Written informed consent was obtained from all individual participants included in the study. Registration of the systematic review was not performed.

### Patient cohort

Patients included in the study were participants in an ongoing longitudinal observational study^20^ (n = 534). The study protocol was approved by the local ethical review board and all participants provided written informed consent. Eligible subjects were those who had a clinical diagnosis of PD (n = 25), MSA (n = 6) or PSP (n = 4) based on consecutive assessments by the same movement disorder specialist, FDG-PET scan performed as a part of their diagnostic investigation, and follow-up of at least two years until June 30th 2019. The most probable diagnosis in the latest medical record annotation was taken into consideration, and all records were reviewed independently by two movement disorders specialists (PS and IM) who were blinded to the results of the FDG-PET automated analysis described below. Sex, age at symptom onset, age at FDG-PET scan, modified Hoehn and Yahr (H&Y) score, comorbidities, dopaminergic medication and other relevant medications were registered. For dopaminergic treatment, levodopa-equivalent daily dose (LEDD) was calculated.

### FDG-PET

All FDG-PET scans were performed in the laboratory of nuclear medicine, Department of Radiology, Karolinska University Hospital Huddinge between November 2012 and June 2019. Patients fasted overnight and oral dopaminergic treatment was not discontinued.

PET scans were performed as 10-min scans, 30–45 min after intravenous injection of 2 MBq/kg weight (min 125 MBq, max 250 MBq). In order to be able to make correction of possible movement artifacts, PET acquisition was done in list mode. Biograph mCT (Siemens) PET-CT with a 21.6 cm FOV was used, providing 148 contiguous 1.47 mm slices. An ultralow dose CT scan (10 mAs) was applied for attenuation correction of PET data. All appropriate corrections, including TOF, were applied and reconstruction was done with OSEM (5 iterations, 21 subsets, 2.0 mm Gaussian filter). Images’ effective resolution was 3 mm.

### Evaluation with automated disease-specific pattern analysis

The applied model, developed at Feinstein Institute, New York, USA, has been previously assessed and replicated^21,22^. FDG-PET scan images were initially inspected for compatibility and quality, with all 35 scans being satisfactory. All images were then pre-processed using statistical parametric mapping (SPM) with the SPM5 software. Using ScAnVp software (available at http://www.feinsteinneuroscience.org) and MATLAB, expression values (z-scores) for PD related metabolic pattern (PDRP), MSARP, and PSPRP were calculated for all patients. These were obtained by, for each voxel, multiplying voxel values and voxel weights, whereupon the sum of all voxels was compared (z-transformed) to a healthy control population of 42 volunteers (mean age 51.6 years, standard deviation (SD) 14.6) with the expression value for each disease pattern being set to 0.0 with a SD of 1.0^16^. From these z-scores, the FDG-PET automated analysis was performed by the imaging experts (DE and CT) blinded to the clinical diagnoses of individual subjects, and the resulting automated differential diagnosis was generated by comparing disease probabilities to the optimal cut-off probabilities established and validated in previous studies^16–18^. At level 1, a differentiation between PD and APS was made. Cut-off probability was > 81% for IPD classification and > 79% for APS classification^16^. Patients not reaching any of these cut-off values (probability for PD < 81% and for APS < 79%) were considered level 1 indeterminate (IND) cases. At level 2, patients classified as APS at level 1 were further classified as either MSA or PSP. Cut-off probability was > 74% for MSA classification and > 55% for PSP classification^16^. Patients not reaching any of these cut-off values (probability for MSA < 74% and < 55% for PSP) were considered level 2 IND cases.

### Statistical analysis

Sensitivity, specificity, positive predictive value (PPV) and negative predictive value (NPV) were calculated using the 2 × 2 table method. The SPM5, ScAnVp and MATLAB analyses of the FDG-PET scans were performed at Feinstein Institute, New York, USA. Statistical analyses were made in Stata version 16.0.

### Meta-analysis

This systematic review was conducted in accordance with the Preferred Reporting Items for Systematic Reviews and Meta-Analyses guidelines^23^ based on a predefined protocol. Two independent reviewers (EP, IM) identified publications of interest by an in-depth search of Medline, Embase, and Web of Science bibliography databases from inception until December 2021, using combination of the following MeSH terms: “FDG-PET”, “Parkinson’s disease-related pattern” and “parkinsonism”. No limitations were set on language or publication year. Initially, articles were selected based on title and abstract, and the full-texts were subsequently obtained and reviewed. Reviewers selected the studies independently and blindly to each other. Reference lists of the eligible studies and relevant reviews were thereafter hand-searched for potentially further studies (“snowball procedure”).

Eligible studies included (a) disease-specific, FDG-PET based pattern analysis of regional glucose metabolism, using scaled subprofile model/principal component analysis to distinguish PD patients from healthy individuals or APS patients including MSA, PSP, with blinded evaluation reported in English; (b) prospective or retrospective, cross-sectional cohort studies that provided sufficient data in order to assess the number of true positive, false negative, true negative, and false positive; and (c) final clinical diagnosis by movement disorders specialists based on clinical criteria^3,24,25^ or on pathology results.

**Figure 1:**
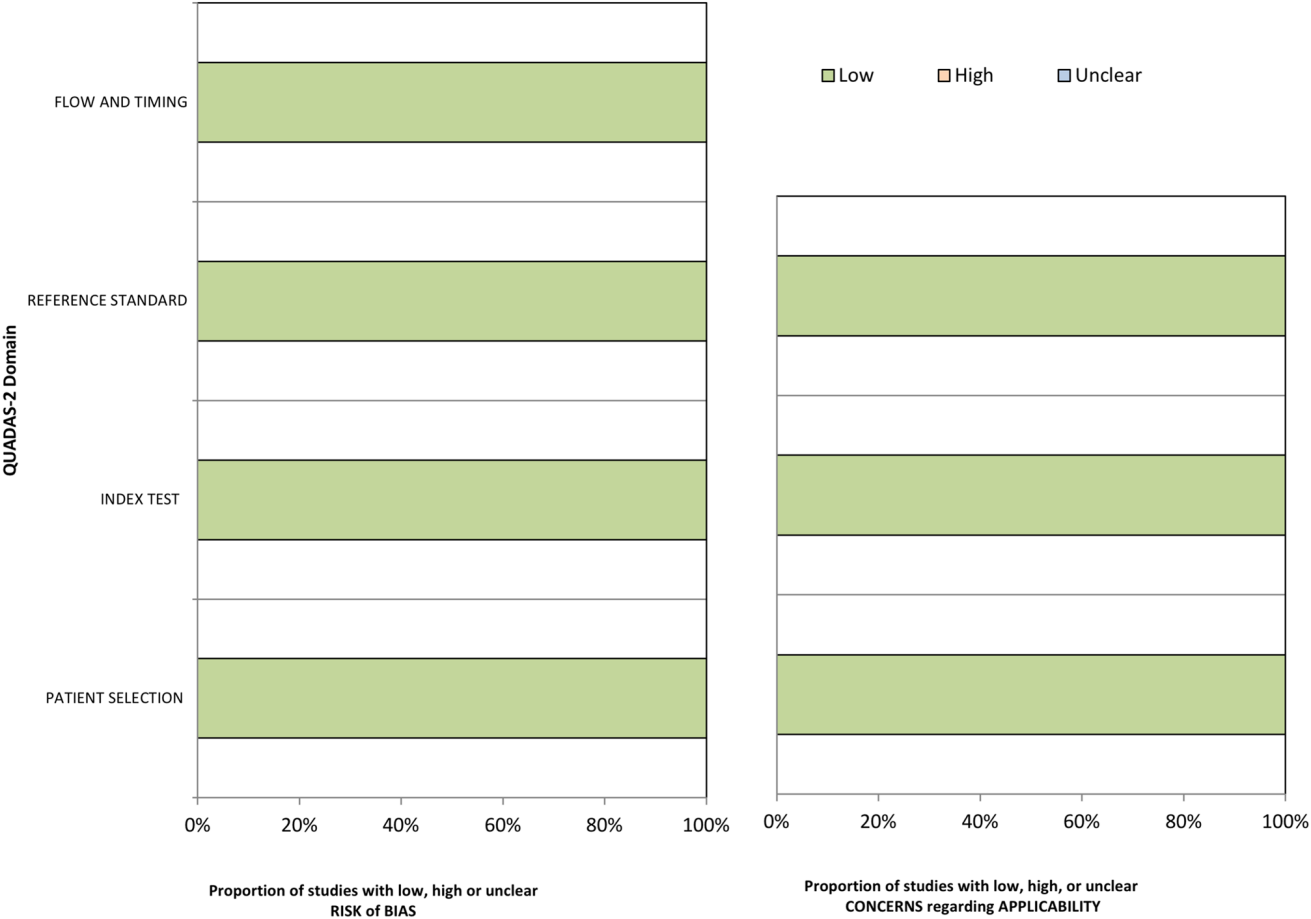
Assessment of risk of bias and concerns regarding applicability of the included studies using the Quality Assessment of Diagnostic Accuracy Studies Tool 2.

General information (year, author, journal, region of origin and study period), patient characteristics (number of patients, mean age, gender, disease duration), ^18^FDG-PET dose and scan time, approaches of metabolic pattern analysis with respective cutoff values, and reference standard for the assessment of the final clinical diagnosis were extracted in a pre-piloted spreadsheet. True and false positive and negative numbers were used for the calculation of diagnostic accuracy. The main outcome measures were the sensitivity and specificity of the automated image-based classification in the discrimination between PD and APS. Secondary outcomes were the sensitivity and specificity of method to distinguish MSA from PSP. The revised tool for the Quality Assessment of Diagnostic Accuracy Studies was used to assessed the quality of the included studies (Fig. 1)^26^. All studies were identified to exhibit a low risk of bias as well as a low risk of concern regarding applicability in all predefined domains.

### Statistical analysis

Pooled sensitivity, specificity, diagnostic odds ratios (DORs) are reported as estimates with 95% confidence intervals (CIs). Hierarchical summary receiver-operating characteristic (sROC) curves were assessed and the area under the curve (AUC) was estimated. Statistical analyses were performed with RevMan 5.4 and R software; packages “mada”, “meta”, “mvmeta” and STATA 16; package “midas”.

## Results

### Patient cohort

The characteristics of our Stockholm cohort are summarized in Table 1. On the level-1 FDG-PET automated analysis, 21 of 25 PD patients and 8 of 10 APS patients (4 MSA and 4 PSP) were classified correctly, and six patients (4 PD and 2 MSA) were classified as indeterminate (IND). Overall, the first-level analysis (Table 2) resulted in 84% sensitivity, 100% specificity, 100% PPV and 71.4% NPV, for the classification of PD and 80% sensitivity, 100% specificity, 100% PPV and 93% NPV for the classification of APS.

**Table 1:** Baseline characteristics of the Stockholm cohort.

Clinical diagnosis	All groups (n = 35)	IPD (n = 25)	MSA (n = 6)	PSP (n = 4)	APS (n = 10)	p-value (IPD vs APS)
**Gender, n (%)**						
Male	16 (46)	12 (48)	3 (50)	1 (25)	4 (40)	0.7
Female	19 (54)	13 (52)	3 (50)	3 (75)	6 (60)	
**Age (y ± SD)**						
At symptom onset	62.0 ± 9.3	61.4 ± 8.6	57.7 ± 10.6	71.8 ± 5.2	63.3 ± 11.2	0.6
At FDG-PET	65.9 ± 10.0	65.4 ± 9.6	61.7 ± 10.6	75.2 ± 6.6	67.1 ± 11.2	0.7
**Symptom duration, n (%)**						
< 2 years	10 (29)	10 (40)	0 (0)	0 (0)	0 (0)	0.03
≥ 2 years	25 (71)	15 (60)	6 (100)	4 (100)	10 (100)	
Symptom duration (y ± SD)	4.2 ± 3.4	4.4 ± 3.3	4.0 ± 2.2	3.5 ± 1.8	3.8 ± 1.9	0.5
mH&Y score	2.7 ± 1.0	2.4 ± 1.0	3.3 ± 0.8	3.8 ± 0.5	3.7 ± 0.7	0.002
**Comorbidities, n (%)**						
Hypertension	13 (37)	5 (20)	2 (33)	3 (75)	5 (50)	0.1
Diabetes	4 (11)	2 (8)	0 (0)	2 (50)	2 (20)	0.6
Dopaminergic medication (n)	30 (86)	22 (88)	6 (100)	2 (50)	8 (80)	0.6
LEDD (mg)	423 ± 306	372 ± 257	753 ± 314	250 ± 300	552 ± 390	0.2
**Other medications, n (%)**						
Antihypertensives	7 (20)	5 (20)	0 (0)	2 (50)	2 (20)	1
Lipid-lowering drugs	6 (17)	2 (8)	1 (17)	3 (75)	4 (40)	0.04
Antidiabetics	4 (11)	2 (8)	0 (0)	2 (50)	2 (20)	0.6
Anticoagulants	7 (20)	2 (11)	2 (33)	3 (75)	5 (50)	0.01

*APS* atypical Parkinsonian syndromes, *FDG-PET* ^18^F-fludeoxyglucose positron emission tomography, *H&Y* modified Hoehn and Yahr score, *IPD* idiopathic Parkinson’s disease, *LEDD* levodopa-equivalent daily dose, *MSA* multiple system atrophy, *PSP* progressive supranuclear palsy, *SD* standard deviation.

**Table 2:** Discriminative measures for the Stockholm cohort.

	Sensitivity	Specificity	PPV	NPV
PD	84% (21/25)	100% (10/10)	100% (21/21)	71.4% (10/14)
APS	80% (8/10)	100% (25/25)	100% (8/8)	93% (25/27)
MSA	50% (2/4)	100% (4/4)	100% (2/2)	67% (4/6)
PSP	50% (2/4)	100% (4/4)	100% (2/2)	67% (4/6)

*APS* atypical parkinsonian syndrome, *MSA* multiple system atrophy, *NPV* negative predictive value, *PD* Parkinson’s disease, *PPV* positive predictive value, *PSP* progressive supranuclear palsy.

After exclusion of the 2 IND cases in level-1, 2 of 4 MSA and 2 of 4 PSP patients were correctly classified in level-2 analysis and two MSA and two PSP were classified as IND APS, resulting in 50% sensitivity and 100% specificity (Table 2).

### Meta-analysis

#### Eligible studies

**Figure 2:**
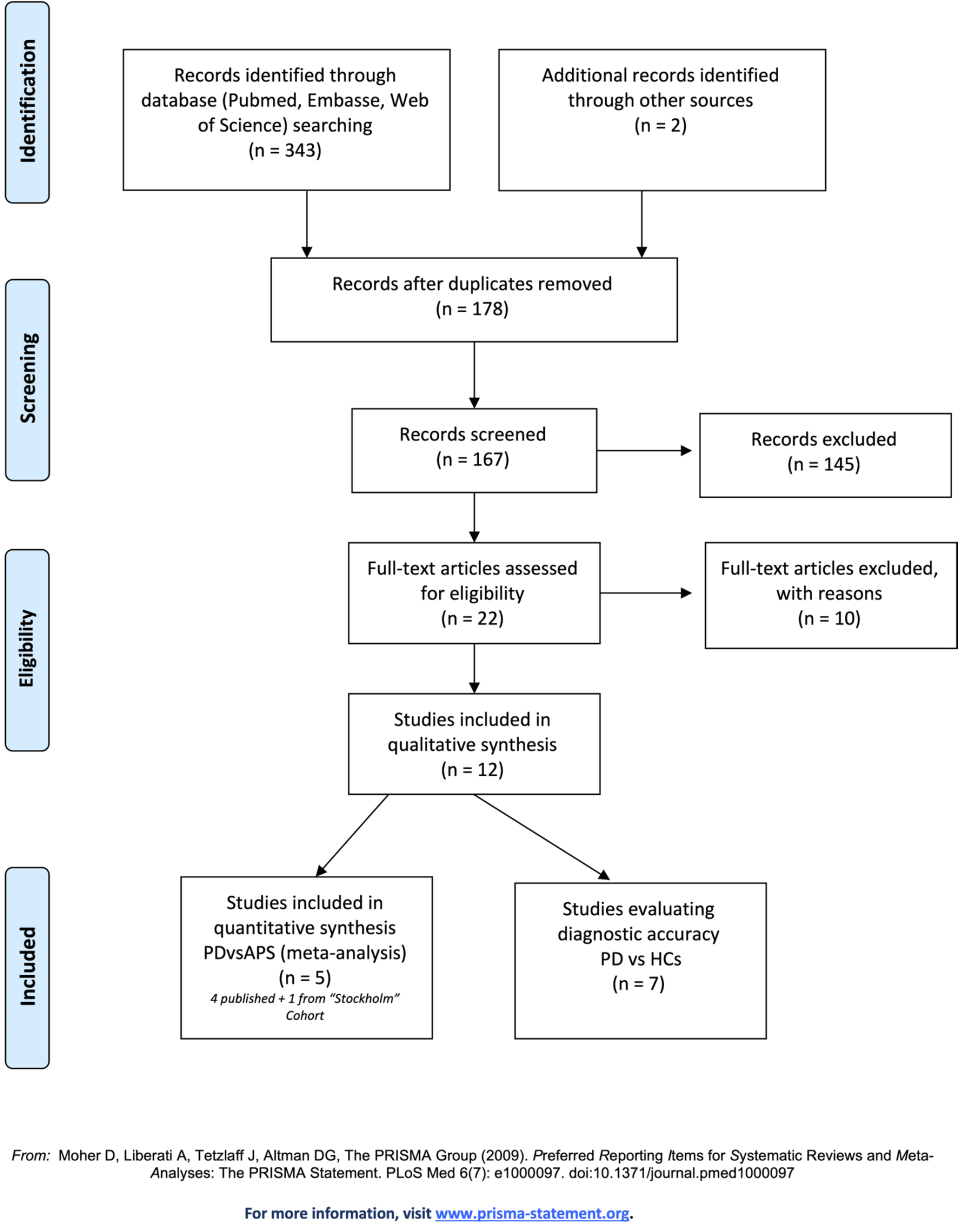
Flowchart of the successive steps of the systematic review process.

Database search yielded 343 studies (Fig. 2), and two additional articles from the “snowball” procedure. At the title and abstract screening level, 145 records were considered irrelevant, and 22 articles were forwarded for full-text evaluation. Subsequently, ten review articles were excluded, leaving 12 studies that were considered for the qualitative synthesis. Because the FDG-PET automated analysis was designed to differentiate among IPD, MSA and PSP patients, but not from healthy controls^16–18^, the reviewers put the focus on the clinical importance and relevance of the differential diagnosis between PD and APS, which led to the decision to exclude studies aiming to evaluate the differentiation of PD from healthy controls (n = 7^27–33^). The final analysis included 5 studies^16–18,34^, including our unpublished data. The characteristics of the studies are summarized in the Table 3. Three studies were conducted in European populations, one in India and one in the USA. Four of the five studies were fully comparable in terms of using the same automated classification algorithm based on disease-related pattern. In total, 492 patients with parkinsonism were investigated. Mean age varied between the studies from 56.2 to 67.1 and symptom duration at diagnosis from 2.7 to 4.95 years. Male sex was predominant in four of five cohorts.

**Table 3:** Characteristics the studies included in the meta-analysis.

Author, year	Country	Study design	Subjects (n)	Sex (% male)	Mean age (years )	Mean disease duration (years)	Scan time (min)	FDG dose (MBq)	Pattern analysis method	Classification algortihm	Reference standard
Tang, 2010^16^	New York, USA	Cohort, PDvsAPD	167	58.7	60.4	5.98	NR	NR	SSM/PCA	Two-level algorithm based on logistic regression of individual patterns scores that quantify expression on specific covariance patterns	Final clinical diagnosis by movement disorders specialist using published clinical diagnosis criteria
Tripathi, 2016	New Delhi, India	Cohort, PDvsAPD	129	69.8	56.1	2.67	20	185–296	SSM/PCA	Two-level algorithm based on logistic regression of individual patterns scores that quantify expression on specific covariance patterns	Final clinical diagnosis by movement disorders specialist using consensus criteria
Rus, 2020	Ljubljana, Slovenia	Cohort, PDvsAPD	56	55.4	67.1	4.06	NR	250	SSM/PCA	Automated two level-algorithm based on the PDRP, MSARP, PSPRP as developed at Feinstein Institute	Clinical diagnosis by movement disorders specialist at least 1 year after FDG-PET, blinded to previous clinical work-up
Marti-Andres, 2020	Pamplona, Spain	Multicenter cohort, PSPvsPD	105	58.9	66.8	2.75	6–15	200	SSM/PCA	Based on the expression of metabolic pattern PSPRP, cutoff Z-score vs. PD patients	Final clinical diagnosis was used as the gold standard
Stockholm Cohort, 2021	Stockholm, Sweden	Cohort, PDvsAPD	35	45.7	65.9	4.2	10	125–250	SSM/PCA	Automated two level-algorithm based on the PDRP, MSARP, PSPRP as developed at Feinstein Institute	All patients enrolled were assessed and investigated by movement disorders specialists

#### Diagnostic accuracy of metabolic patterns

**Figure 3:**
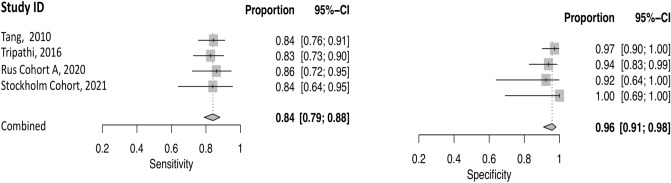
Forest plot of the included studies presenting sensitivity and specificity of each study along with the combined measures—first level of classification model, PD vs APS.

**Figure 4:**
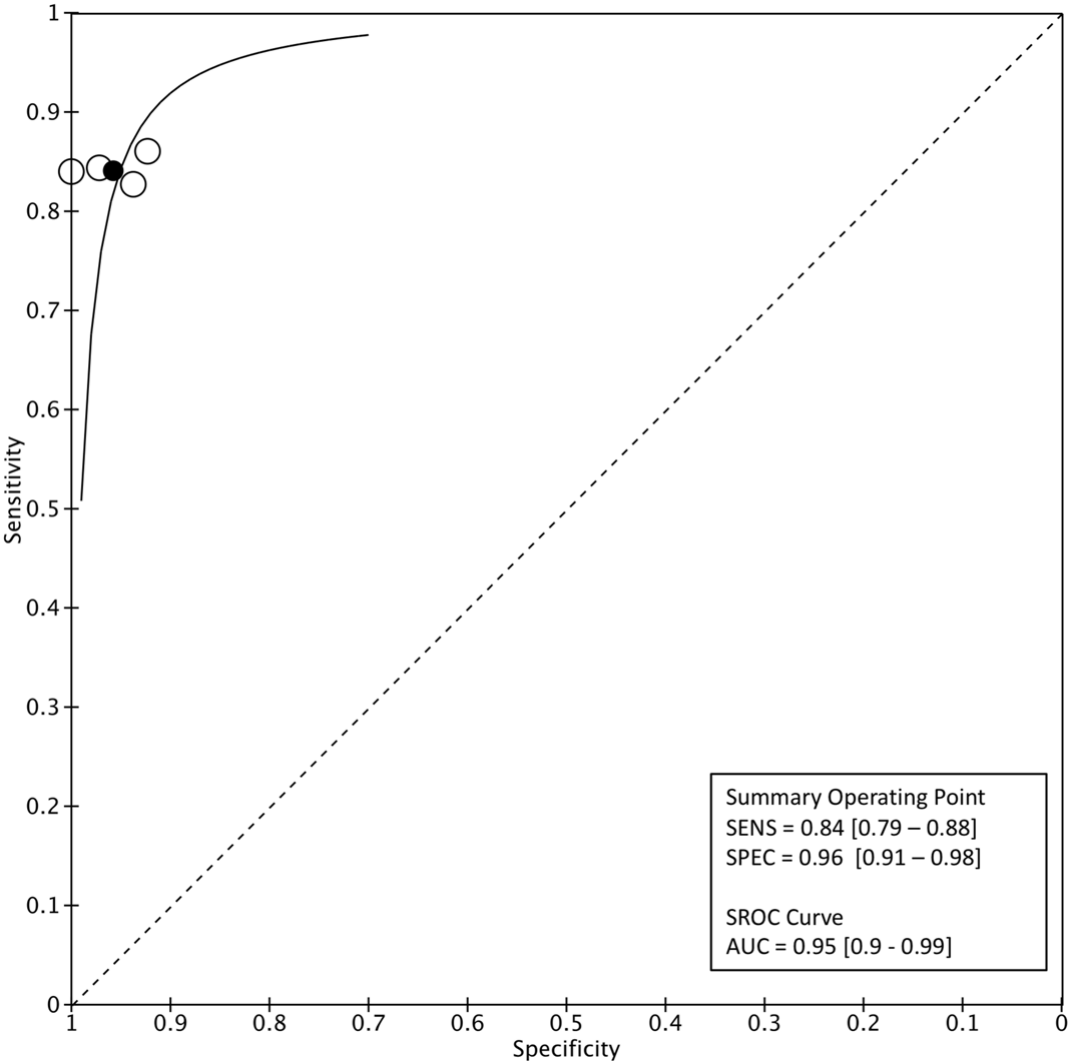
Level-1 classification algorithm for PD: Summary ROC plot with mean operating sensitivity and specificity point.

The pooled sensitivity for IPD vs. APS in the first classification level was 0.84 (95% CI 0.79–0.88), and the pooled specificity was 0.96 (95% CI 0.91–0.98). Positive Likelihood Ratio (PLR) was 19.9 (95% CI 9.1–43.6), Negative Likelihood Ratio (NLR) 0.17 (95% CI 0.12–0.22) and the Diagnostic Odds Ratio (DOR) 119.7 (95% CI 49.3–290.4). Forest plot is displayed in Fig. 3. Hierarchical sROC curve indicated that the AUC was 0.95 (95% CI 0.9–0.99), illustrating high discriminating ability (Fig. 4). As the studies followed identical methods, no heterogeneity was observed.

**Figure 5:**
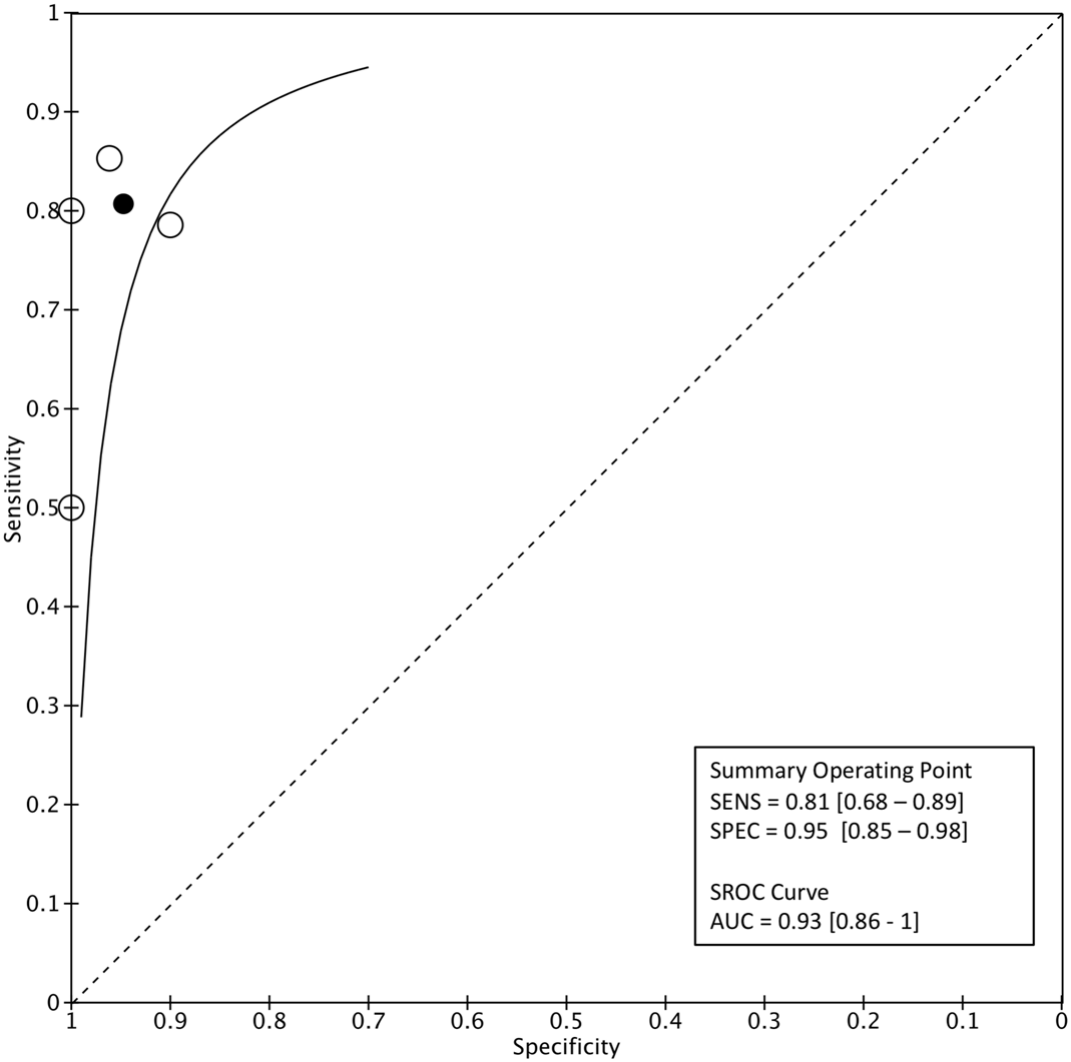
Level-2 classification algorithm for MSA: Summary ROC plot with mean operating sensitivity and specificity point.

**Figure 6:**
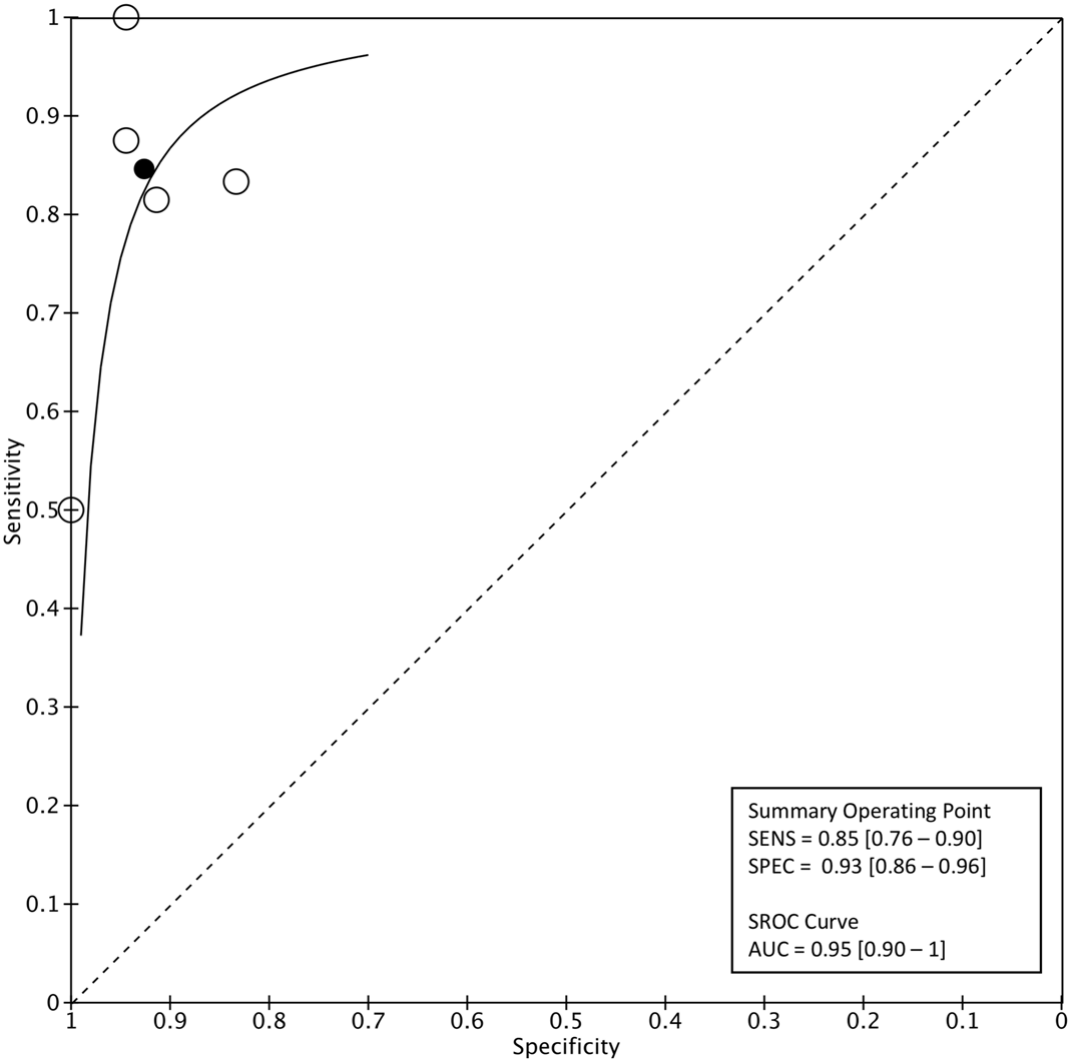
Level-2 classification algorithm for PSP: Summary ROC plot with mean operating sensitivity and specificity point.

At the level-2 classification analysis, sensitivity for MSA was 0.81 (95% CI 0.68–0.89) and specificity 0.95 (95% CI 0.85–0.98). PLR was 15.3 (95% CI 5–46.4), NLR was 0.2 (95% CI 0.1–0.3) and the DOR was 75.3 (95% CI 19.8–286.2). Based on sROC, the AUC for MSA was 0.93 (95% CI 0.86–1). (Fig. 5). For the classification of PSP, the pooled sensitivity was 0.85 (95% CI 0.76–0.90) and specificity 0.93 (95% CI 0.86–0.96). PLR was 11.4 (95% CI 6.08–21.6), NLR 0.17 (95% CI 0.1–0.26) and DOR 69.05 (95% CI 29.1–163.7). sROC curve indicated that the AUC was 0.95 (95% CI 0.90–1; Fig. 6).

## Discussion

We report that the FDG-PET based automated classification algorithm previously developed^16^ to measure disease-specific metabolic patterns and distinguish between parkinsonian syndromes, has been replicated in 35 Swedish patients with PD, MSA and PSP with very good accuracy. We also performed a meta-analysis that showed that automated disease-specific patterns method have excellent specificity and very good sensitivity for all three diagnoses.

In our patient cohort the algorithm could accurately differentiate between PD and APS. Differentiation between MSA and PSP was less accurate, however no misdiagnosis occurred (100% specificity). PD classification precision was in line with the results of the conducted meta-analysis that included data from almost 500 subjects, with 84% sensitivity and 96% specificity for the classification of PD vs. APS, thus confirming very high diagnostic accuracy. Three patients in our cohort had probabilities that were 2, 3 and 1 percentage points under the threshold. These cases, that are very close to the threshold for one diagnosis and have very low probability for the alternative diagnosis, may be managed less rigidly in clinical praxis, thus indicating even higher accuracy of the method in real-life applications. Based on these results, FDG-PET combined with automated classification algorithm may be considered in routine clinical care to aid earlier diagnosis, especially in cases movement disorders specialists are not available. Additionally, application of the automated algorithm in clinical trials may be of value, in order to increase homogeneity in patient selection with regard to network activity pattern^17^.

Patients included in the meta-analysis had varying symptom duration at diagnosis, from 2.7 to 4.95 years, with two studies^16,18^ presenting subgroup analysis for patients with symptom duration less than 2 years. In both studies the classification algorithm had excellent PPV underlining its value in early and accurate PD diagnosis. Reliable prognostic counselling and mobilization of resources can be significantly accelerated with increased and earlier diagnostic certainty^35^. Moreover, selection of patients in early stages of the disease course in clinical trials of potentially disease-modifying drugs can be improved^36,37^. Importantly, PDRP has recently been reported to be expressed in early-stage, treatment-naive PD patients^38^.

Neuroimaging biomarkers have been discussed for the diagnostic work up of parkinsonism but until now, only conventional MRI and ultrasonography have been established according to the international criteria, whereas the role of FDG-PET is still to be determined^39^. Different analytical approaches of the FDG-PET patterns have been validated. Notably, the diagnostic accuracy of FDG-PET depends on the operating procedures^40^. In a recent meta-analysis^41^, observer-dependent and observer-independent methods using metabolic imaging were very accurate (> 90%) in distinguishing PD from APS—as long as the observers were highly experienced. In this context, neuroimaging methods incorporating automated algorithms remain the most promising method for a reliable and widely available diagnostic tool.

Our study has limitations that should be considered in result interpretation, including the small sample size of the patient series and the lack of pathological confirmation as the diagnostic gold standard. However, our results were well in accordance with previous larger studies and contributed to the meta-analysis. Nevertheless, including our results in this small, though clinically meaningful, meta-analysis we provide robust evidence on the diagnostic accuracy of this automated classification method based on FDG-PET metabolic patterns. Finally, our Stockholm-cohort adds further evidence on the generalizability and consistency of the method that produces similar results across geographic regions and patient populations.

In conclusion, our results indicate that FDG-PET based network analysis in combination with an automated probability-based algorithm may be successfully applied in the differential diagnosis in early stages of parkinsonism.

## Data Availability

The datasets generated and/or analyzed during the current study are available from the corresponding author on reasonable request.
